# RAMPA Therapy: Effects on Craniofacial Growth Assessed by Coben Analysis and Statistical Evaluation

**DOI:** 10.3390/jcm15051882

**Published:** 2026-03-01

**Authors:** Yasushi Mitani, Yuko Okai-Kojima, Takahisa Shimazaki, Mohammad Moshfeghi, Morio Tonogi, Shouhei Ogisawa, Bumkyoo Choi, Mitsuru Motoyoshi

**Affiliations:** 1 Codomo Clinic, Tokyo 180-0004, Japan; mitani@trust.ocn.ne.jp; 2 Children and Women Dental Clinic, Tokyo 106-0046, Japan; yukoyukono@gmail.com; 3 Shimazaki Orthodontic Office, Ibaraki 316-0013, Japan; takahisa.shimazaki@gmail.com; 4 Department of Mechanical Engineering, Sogang University, Seoul 04107, Republic of Korea; mmoshfeghi@sogang.ac.kr; 5 Department of Oral & Maxillofacial Surgery, School of Dentistry, Nihon University, Tokyo 101-8310, Japan; tonogim@gmail.com (M.T.); ogisawa.shouhei@nihon-u.ac.jp (S.O.); 6 Yokohama Clinic, Department of Advanced Oral and Maxillofacial Surgery, Kanagawa Dental University, Yokohama 221-0835, Japan; 7 Department of Orthodontics, Nihon University School of Dentistry, Tokyo 101-8310, Japan; motoyoshi.mitsuru@nihon-u.ac.jp

**Keywords:** RAMPA, maxillary hypoplasia, skeletal Class III malocclusion, Coben analysis, craniofacial growth, retrospective cohort study

## Abstract

**Objective:** This retrospective comparative cohort study investigated the craniofacial growth effects of the RAMPA (Right Angle Maxillary Protraction Appliance) system. The system aims to protract the maxilla in an anterosuperior direction to address maxillary hypoplasia and skeletal Class III malocclusion, potentially mitigating the posteroinferior displacement often associated with conventional orthopedic approaches. **Materials and Methods:** Craniofacial measurements were analyzed before (T1) and after (T2) RAMPA treatment in 30 growing patients (17 males, mean age 7.32 years; 13 females, mean age 8.34 years). Coben analysis was utilized to quantitatively evaluate coordinate relationships and proportional changes based on the Frankfurt Horizontal plane. Statistical significance was determined using paired *t*-tests or Wilcoxon signed-rank tests (two-sided, α = 0.05) without adjustment for multiple comparisons due to the exploratory nature of the study. **Results:** RAMPA treatment was associated with significant increases in facial depth (Ba-N) and anterior facial height (N-Me) in both sexes. Specifically, facial depth increased by an average of 2.65 mm in males (*p* = 0.001) and 2.18 mm in females (*p* = 0.007). Female patients showed a significant increase in the maxillary depth ratio (Ptm-A/Ba-N), while males exhibited a significant decrease in the Gonial Angle (avg. 1.47° decrease), suggesting anterior mandibular rotation. **Conclusions:** RAMPA treatment effectively promoted anterosuperior craniofacial growth and induced favorable mandibular rotation in this cohort. These findings suggest the system has potential clinical value for improving craniofacial balance in skeletal Class III malocclusion. While improved cervical posture is a theoretical benefit of such remodeling, systemic outcomes were not directly measured in this study.

## 1. Introduction

Skeletal Class III malocclusion, arising from maxillary hypoplasia, mandibular overgrowth, or a combination of these factors, represents a significant challenge in orthodontics. It can lead not only to esthetic concerns but also to functional impairments in mastication and speech [1,2]. Especially in growing patients with Class III malocclusion, early diagnosis and appropriate orthopedic treatment are crucial to reduce the need for surgical intervention in adulthood, aiming to establish a stable occlusal relationship by favorably modifying jaw growth patterns [3,4]. Recent epidemiological reviews confirm that skeletal Class III malocclusion remains globally prevalent, reinforcing the clinical importance of effective early orthopedic intervention during growth [5].

One of the traditional treatment methods for Class III malocclusion, involving Rapid Maxillary Expansion (RME) combined with a facemask, has been proven effective in inducing anterior movement of the maxilla [6,7,8,9,10]. However, these methods have often been noted for limitations such as inducing posteroinferior displacement of the maxilla and increasing clockwise rotation of the mandible, which can lead to an increase in lower facial height and labial proclination of the maxillary incisors [3,10,11,12,13]. These limitations have persisted in terms of long-term stability and facial esthetics of treatment outcomes [1], and despite attempts to use bone-anchored maxillary protraction [3], further research on vertical control is still needed. Recent systematic and umbrella reviews published after 2017 have consistently confirmed these limitations, reporting persistent challenges in vertical control and mandibular rotational side effects despite advances in skeletal anchorage [14,15,16]. A recent systematic review reported that bone-anchored maxillary protraction (BAMP) achieves significantly greater skeletal advancement with superior vertical control and reduced dentoalveolar compensation compared with conventional facemasks or observation in children under 12 years [17]. Clinical evidence further supports the importance of controlled force vectors in orthopedic treatment. A comparative study evaluating different intermaxillary elastic polymers in growing Class II patients demonstrated that altering elastic force magnitude and replacement intervals produced significantly different skeletal responses, with more frequent elastic replacement yielding a greater increase in the SNB angle (*p* < 0.05). Although focused on Class II correction, the study highlights a broader biomechanical principle: craniofacial growth modification is highly sensitive to the direction and consistency of applied forces. This reinforces the need for orthopedic systems capable of delivering stable, well-oriented vectors during growth [18].

**Figure 1:**
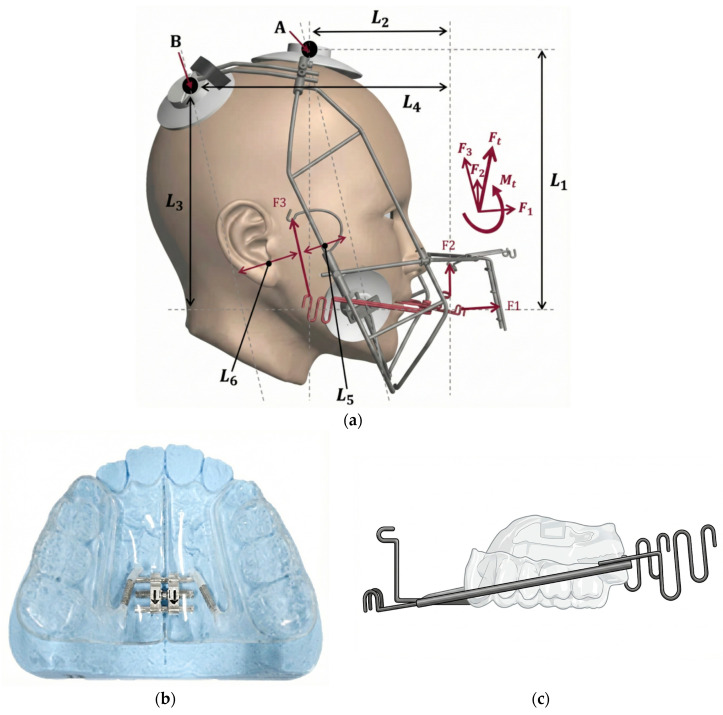
RAMPA system; (**a**) RAMPA (extraoral appliance) mounted on the face, (**b**) intraoral appliance, (**c**) connecting bar (active bow) between the intraoral and extraoral appliances.

The Right Angle Maxillary Protraction Appliance (RAMPA) is an extraoral device that can be combined with different intraoral devices (Figure 1a–c). RAMPA aims to protract the maxilla in an anterosuperior direction, counteracting gravity, which is expected to establish correct occlusion and positively influence craniofacial growth in growing patients. The RAMPA system consists of an intraoral appliance connected to an active bow, which applies a combination of horizontal force (F1), anterior vertical force (F2), and posterior force (F3) to generate a total anterosuperior force (Ft) and a total counter-clockwise moment (Mt), designed to induce not only simple translation but also rotation of both the maxilla and mandible. This precise design is supported by previously published Finite Element Method (FEM) simulation and clinical results, providing the biomechanical basis for RAMPA to induce specific skeletal changes. However, no clinical study has quantitatively evaluated whether RAMPA-induced vector control translates into proportional craniofacial growth modulation when assessed using a coordinate-based analytical system.

Recent biomechanical analyses emphasize that successful orthopedic growth modulation depends critically on precise control of force vectors and generated moments, rather than force magnitude alone [19]. To overcome the limitations of existing treatments and to facilitate more biomechanically favorable growth modulation, the RAMPA (Right Angle Maxillary Protraction Appliance) system was devised and proved to provide promising correction to anterosuperior protraction during maxillary expansion [20,21,22,23].

Accordingly, contemporary studies advocate three-dimensional and proportional analytical approaches to capture craniofacial growth changes beyond conventional angular measurements [24,25].

Additionally, three-dimensional Cone-Beam Computed Tomography (CBCT)-based analyses have emphasized the importance of precise spatial assessment in craniofacial correction, as demonstrated by Deepal Haresh Ajmera et al., who identified residual asymmetry patterns even after orthognathic surgery, underscoring the necessity of comprehensive 3D evaluation [26].

The craniofacial complex exhibits complex growth patterns involving the interaction of various components [27], with significant individual variations, especially during puberty. To clearly understand and quantitatively analyze these complex growth patterns, Coben analysis is utilized. Coben analysis quantitatively analyzes craniofacial morphology through coordinate relationships of depth and height, contributing to a clear understanding of proportional changes in each component that were difficult to ascertain with conventional angular measurement methods [28]. It is considered a valid method capable of evaluating not only static morphology but also the dynamic changes in growth and development. This analysis method, by focusing on proportional changes within the craniofacial complex, becomes an important tool for understanding the effects of orthopedic appliances and evaluating overall growth pattern changes.

This study quantitatively validated the actual clinical effects of the RAMPA system through Coben analysis, designed to minimize the posteroinferior displacement of the maxilla and clockwise mandibular rotation (i.e., drawbacks of conventional RME/facemask treatment). Instead, the current study focuses on RAMPA that results in anterosuperior maxillary protraction and counter-clockwise mandibular rotation.

This study provides clinical data supporting RAMPA’s potential biomechanical advantages in inducing jaw growth against gravity, beyond simple anterior translation. While a considerable number of previous studies primarily focused on the effects of facemasks [1,6,7,9,10], our in-depth analysis of the clinical effects of an appliance like RAMPA, which attempts 3D growth control through specific vector forces using Coben analysis, is a significant differentiating factor of this study. It actively utilized Coben analysis not merely for morphological assessment but for evaluating the dynamic changes in growth and development, particularly the proportional changes in each component. It should be mentioned that while Cone-Beam Computed Tomography (CBCT) provides excellent 3D evaluation, we utilized Coben analysis on 2D lateral cephalograms to minimize radiation exposure in this young, growing cohort, and to allow for direct, standardized proportional comparisons with the extensive historical 2D natural growth database established by Hideo Mitani [29]. Specifically, by statistically analyzing various depth and height proportional changes before and after RAMPA treatment, this study clearly presented subtle patterns of jaw growth modulation that were difficult to identify with conventional angular measurement methods. Furthermore, by comparatively reviewing Hideo Mitani’s natural growth study data based on Coben analysis, this study clearly demonstrated how RAMPA treatment-induced growth changes interact with and differentiate from natural growth patterns, rather than simply promoting growth. This quantitatively highlights the specific effects of the treatment, such as promoting anterosuperior growth consistent with natural growth directions while inducing stronger anterior maxillary growth than natural development. While most studies limit comparisons to treated vs. untreated (control) groups, this study deepened the clinical significance of the treatment effects through comparison with a long-term, universal criterion: natural growth. Such growth redirection has also been reported to influence upper airway dimensions, suggesting potential functional implications of growth-directed orthopedic treatment in growing patients [30]. It should be mentioned that although previous literature suggests that growth redirection may influence upper airway dimensions, the present study did not directly measure airway volume or cervical posture.

## 2. Materials and Methods

### 2.1. Study Design and Patient Sample

This investigation employed a retrospective comparative cohort study design, analyzing data from patients who had previously undergone RAMPA therapy. Ethical approval was obtained from the Ethics Committee of Nihon University School of Dentistry (EP22D001).

The sample consisted of 30 growing patients diagnosed with skeletal Class III malocclusion characterized by maxillary hypoplasia. The cohort included 17 males (mean age 7.32 ± 0.86 years) and 13 females (mean age 8.34 ± 1.24 years).

- Inclusion Criteria: (1) Diagnosis of skeletal Class III malocclusion with maxillary deficiency; (2) Mixed dentition stage; (3) Pre-peak skeletal maturity; (4) Good compliance with appliance wear.
- Exclusion Criteria: (1) Presence of congenital craniofacial syndromes (e.g., cleft lip/palate); (2) History of prior orthodontic or orthopedic treatment; (3) Severe systemic disease.

### 2.2. Treatment Protocol

All patients were treated with the RAMPA system (Figure 1a–c), consisting of an intraoral appliance (modified expander) connected to an extraoral active bow.

- Force System: The system applied a combination of horizontal (F1), anterior vertical (F2), and posterior (F3) forces. The force ratio was F1:F2:F3 = 2:1:3, based on previous research [20], to generate a total anterosuperior force (Ft) and counter-clockwise moment (Mt) in forward rotation.
- Duration: The average treatment duration (T1 to T2) was approximately (1.5 ± 0.7) years.
- Concurrent Therapy: The intraoral unit was activated for expansion once every two weeks.

### 2.3. Coben Analysis

**Figure 2:**
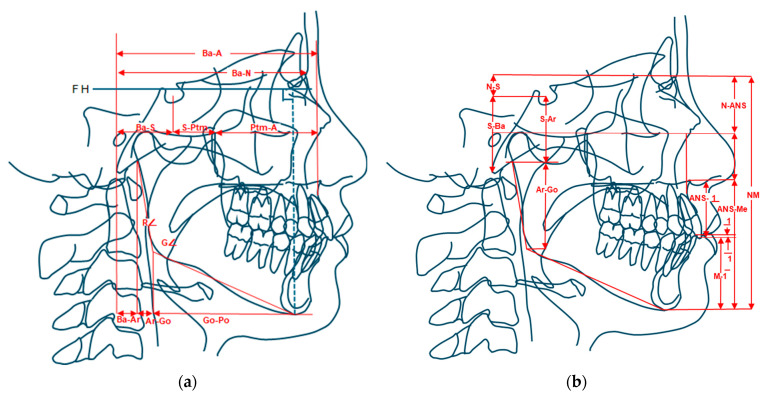
Cephalometric landmarks and measurements used in Coben’s proportional analysis. (**a**) Horizontal measurements (Ba-N, Ba-A, Ba-S, S-Ptm, Ptm-A, Ba-Ar, Ar-Go, Go-Po, Ramus inclination, and Gonial angle); note the perpendicular line derived from Pogonion (Po) to clearly define the mandibular depth limit. (**b**) Vertical measurements (N-Me, N-S, S-Ba, S-Ar, Ar-Go, N-ANS, ANS-Me, ANS-1, Overbite, and M-1).

Lateral cephalograms were obtained at T1 (pre-treatment) and T2 (post-treatment). Coben analysis was used to evaluate craniofacial morphology (Figure 2a,b).

- Landmark Identification: Cephalometric tracings were performed by Dr. Takahisa Shimazaki.
- Reliability: To assess the reliability of the measurements, 10 randomly selected cephalograms were re-traced and re-measured by the same examiner (intra-examiner reliability) and by a second examiner (inter-examiner reliability). Key craniofacial parameters representing horizontal and vertical dimensions (e.g., Ba-N, N-Me, Ptm-A, and Gonial Angle) were selected for the reliability assessment. The resulting Intraclass Correlation Coefficients (ICCs) for these measurements were all above 0.90, indicating excellent reliability and reproducibility of the Coben analysis.
- Measurements: Depth (anteroposterior) and height (vertical) coordinates were measured relative to the Frankfurt Horizontal (FH) plane and a perpendicular vertical axis. Proportions were calculated relative to total facial depth (Ba-N) and height (N-M). To ensure clarity in Figure 2a, the mandibular measurement ranges are defined by lines perpendicular to the measurement axis derived from relevant landmarks (e.g., Pogonion).

### 2.4. Statistical Analysis

Statistical analysis was conducted using R software (v. 4.3.3).

- Normality Test: The Shapiro–Wilk test checked the normality of differences (T2−T1).
- Hypothesis Testing: Paired *t*-tests were used for normally distributed variables; Wilcoxon Signed-Rank tests were used for non-normally distributed data. All tests were two-sided with a significance level of α = 0.05.
- Adjustment: No correction for multiple comparisons (e.g., Bonferroni) was applied due to the exploratory nature of this study.

Coben analysis provides a precise methodology for the quantitative evaluation of craniofacial complex morphology.

1. Reference Planes and Coordinates: The Frankfurt Horizontal Plane (FH) serves as the transverse coordinate axis for measuring craniofacial depth, and a plane perpendicular to the FH plane serves as the longitudinal coordinate axis for measuring height. The distance of each anatomical area is measured as depth or height based on this coordinate system.
2. Proportional Analysis: The measured depth and height of each area are expressed as percentages (%) relative to the total facial depth (Nasion-Basion, Ba-N) and total facial height (Nasion-Menton, N-M). This proportional analysis provides essential information for understanding how each component contributes to overall growth and how inter-component relationships change.

Key Measurement Categories:

- Depth (Anteroposterior Length): These measurements are projections onto the FH plane. The total facial depth (Ba-N) is set as 100%. Measurements include the posterior cranial base proportion (Ba-S/Ba-N), middle face proportions (e.g., Ptm-A/Ba-N for maxillary depth and Ba-A/Ba-N for midfacial depth), and lower face proportions (e.g., Ba-Pog/Ba-N, Ar-Go/Ba-N, Ar-Pog/Ba-N).
- Height (Vertical Length): These measurements are projections onto a plane perpendicular to the FH plane. The vertical reference unit is Nasion-Menton (N-M). Measurements include posterior facial height proportions (e.g., S-Ar/N-M, Ar-Go/N-M) and anterior facial height proportions (e.g., N-ANS/N-M, ANS-M/N-M).

Mandibular Metrics: The height of the mandibular ramus (Ar-Go), the length of the mandibular body (Go-Pog), and the inclination angle of the mandibular plane (∠MPI) are also examined, alongside the Gonial Angle (∠Go), to evaluate their relationship with the cranial base and rotational effects.

## 3. Results

The statistical analysis results detail the changes in Coben measurements for the male and female cohorts, confirming the expected growth induction patterns aligned with the RAMPA device’s design purpose.

### 3.1. Male Patient Analysis Results (n = 17)

The statistical analysis results for the 17 male patients are presented in Table 1.

- Overall Growth Indicators: Significant increases were observed in key linear measurements, including facial depth (Ba-N) (avg. 2.65 mm increase) and anterior facial height (N-Me) (avg. 3.97 mm increase). This directly reflects the intended anterosuperior growth induction effect.
- Cranial Base and Maxilla: A significant increase was found in the posterior cranial base (Ba-S) and its ratio to total facial depth (Ba-S/Ba-N). A significant increase in the maxillary depth ratio (Ptm-A/Ba-N) was also observed, indicating desirable proportional skeletal remodeling and effective anterior maxillary protraction.
- Mandibular Rotation and Rearrangement: A significant decrease in the Gonial Angle (avg. 1.47° decrease) suggested anterior rotation (counter-clockwise) and a more horizontal growth pattern of the mandible. Furthermore, the mandibular ramus height-to-depth ratio (Ar-Go/Ba-N), mandibular length-to-depth ratio (Ar-Pog/Ba-N), and lower facial depth ratio (Ba-Pog/Ba-N) all significantly decreased relative to total facial depth (Ba-N). This signifies a complex 3D rearrangement, where anterior rotation causes these linear measurements to appear proportionally smaller against the fixed Coben reference plane.

### 3.2. Female Patient Analysis Results (n = 13)

The statistical analysis results for the 13 female patients are presented in Table 2.

- Overall Growth Indicators: Similarly to male patients, significant increases were observed in overall growth indicators, facial depth (Ba-N) (avg. 2.18 mm increase), and anterior facial height (N-Me) (avg. 3.64 mm increase). This confirms that RAMPA effectively induces anterosuperior growth regardless of gender.
- Maxillary Protraction (Strong Effect): Particularly strong proportional increases were observed in the maxillary depth ratio (Ptm-A/Ba-N) and the midfacial depth ratio (Ba-A/Ba-N). This suggests that RAMPA’s primary goal of anterior maxillary protraction was strongly expressed in female patients.
- Mandibular Influence: A significant proportional decrease was observed in the mandibular ramus height/facial height ratio (Ar-Go/N-M), suggesting that the rotational effect on the mandible also occurred in females.

### 3.3. Comparison with Natural Growth Patterns Based on Hideo Mitani’s Study

Craniofacial growth changes observed with RAMPA treatment show both similarities and differences when compared to Hideo Mitani’s [28] longitudinal observation of natural growth patterns in Japanese individuals aged 7 to 15 years. These comparisons are described in Table 3.

**Figure 3:**
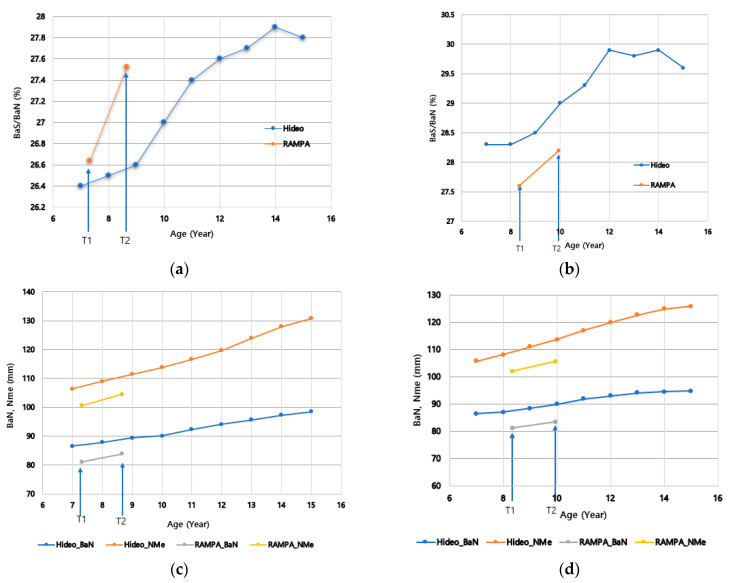

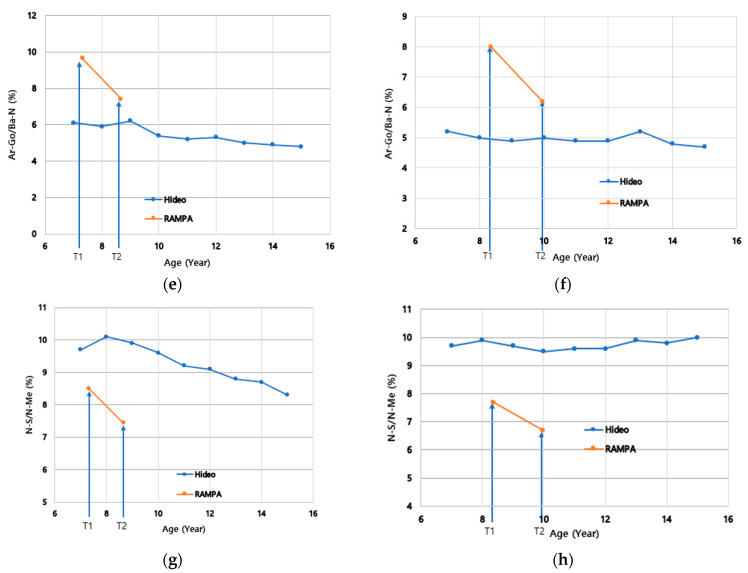
Comparison of craniofacial growth changes between the RAMPA treatment group and the natural growth control group. (Both datasets are presented as solid lines, with RAMPA representing the mean changes in the treated cohort and Hideo indicating the natural growth patterns reproduced from the historical data of Hideo Mitani [29]. The *y*-axis represents the magnitude of change in millimeters (mm) or percentage (%), as indicated for each parameter, and the *x*-axis indicates the age in years. Note that the natural growth lines are derived from a different chronological cohort (Mitani [29]), and no formal statistical comparison was performed between the two datasets due to differences in sample characteristics and time frames. (**a**) Mandibular depth ratio (Ba-S/Ba-N) in males, (**b**) Mandibular depth ratio (Ba-S/Ba-N) in females, (**c**) Facial depth (Ba-N) and anterior facial height (N-Me) in males, (**d**) Facial depth (Ba-N) and anterior facial height (N-Me) in females, (**e**) Ramus height-to-depth ratio (Ar-Go/Ba-N) in males, (**f**) Ramus height-to-depth ratio (Ar-Go/Ba-N) in females, (**g**) Anterior facial height-to-depth ratio (N-S/N-Me) in males, (**h**) Anterior facial height-to-depth ratio (N-S/N-Me) in females).

- Midfacial Growth Differentiation: Hideo Mitani’s [28] natural growth study showed that the midfacial depth ratio (Ba-A/Ba-N) remained almost constant, and the superior alveolar base depth ratio (Ptm-A/Ba-N) tended to decrease slightly relative to total depth. In contrast, RAMPA treatment induced significant and highly significant increases in the maxillary depth ratio (Ptm-A/Ba-N) in both sexes.
- Anterosuperior Enhancement: RAMPA treatment promoted anterosuperior growth consistent with natural growth directions. However, the treatment exhibited a differentiated effect, inducing anterior maxillary growth more powerfully than natural development.
- Mandibular Modulation: While natural growth primarily involves significant depth growth of the mandibular body (Go-Pog), RAMPA modulated this pattern by inducing favorable anterior rotation of the mandible. The slope of the treatment effect during the RAMPA therapy period, as illustrated in Figure 3a–h, is steeper compared to that of natural growth, demonstrating the certainty and potency of the treatment’s effectiveness.

## 4. Discussion

### 4.1. Biomechanical Effects and Clinical Implications

The results indicate that the RAMPA system generates anterosuperior protraction forces, evidenced by the consistent increase in facial depth and height across genders. This suggests the appliance may help mitigate the adverse posteroinferior rotation often seen in Class III treatments. The significant decrease in the Gonial Angle in males supports the hypothesis that the applied force vector (Mt) can induce counter-clockwise mandibular rotation. These findings align with recent studies on the efficiency of intermaxillary elastic polymers in orthopedic treatments, highlighting the importance of force vector control.

### 4.2. Age and Gender Considerations

We observed differences between genders, notably the significant Gonial Angle decrease in males but not females. Part of this variation may relate to the age difference between the cohorts (males: 7.32 years; females: 8.34 years) and the generally earlier onset of skeletal maturation in females. Although the female cohort is still within the early mixed-dentition stage, this one-year age difference may nonetheless act as a confounding factor influencing growth responsiveness. It may also partially contribute to the reduced mandibular rotational change observed in females, alongside inherent sexual dimorphism in craniofacial growth timing and biomechanical response.

### 4.3. Systemic Health Hypotheses

While this study focused on skeletal changes, the redirection of craniofacial growth may theoretically influence the upper airway and cervical posture. However, as this study did not include airway measurements or posture analysis, any systemic benefits remain hypothetical and require further investigation.

The major craniofacial structural changes observed across both cohorts and their associated clinical and biomechanical implications are synthesized in Table 4.

### 4.4. Limitations

1. Lack of Concurrent Control: The primary limitation is the absence of a concurrent untreated Class III control group or an active comparison group (e.g., conventional facemask). Comparisons were made against historical natural growth data, which does not account for secular trends or specific selection bias.
2. Sample Size: The sample size (n = 30) is relatively small, and the study was likely underpowered for some secondary variables.
3. Retrospective Design: The retrospective nature introduces potential selection bias.
4. Generalizability: As the cohort consists of Japanese patients, findings may not be directly generalizable to other ethnic groups with different craniofacial norms.
5. This study involved numerous statistical tests across many craniofacial parameters. Because the analysis was exploratory, no adjustment for multiple comparisons (e.g., Bonferroni correction) was applied. This increases the risk of Type I error, and therefore, individual *p*-values should be interpreted with caution. The decision was intentional to avoid inflating Type II error and to identify potential growth modulation trends that warrant confirmation in future hypothesis-driven studies. In addition, analyses were conducted separately for males and females due to well-established sexual dimorphism in craniofacial growth timing and peak growth velocity; pooling the data would have risked masking these physiologically distinct growth responses.

## 5. Conclusions

Within the limits of this retrospective study, the results suggest that RAMPA therapy is effective in promoting anterosuperior craniofacial growth and inducing favorable anterior mandibular rotation in growing patients with skeletal Class III malocclusion. The quantitative evaluation using Coben analysis indicates that the system appears to offer biomechanical advantages in vertical control compared to historical natural growth patterns. However, given the limitations of the sample size and study design, these findings should be interpreted with caution. Future prospective studies, ideally randomized controlled trials with concurrent control groups, are necessary to definitively confirm its clinical superiority over conventional orthopedic approaches and to substantiate the potential systemic health benefits.

## Figures and Tables

**Table 1:** Comparison of Coben analysis measurements before (T1) and after (T2) RAMPA treatment in male patients (*n* = 17).

Category and Measurement	T1 Mean ± SD	T2 Mean ± SD	Mean Diff.	*p*-Value	Statistical Test
Cranial Base and Depth					
Ba-N (mm)	81.09 ± 4.57	83.74 ± 5.69	2.65	0.001 *	Paired *t*-test
Ba-S (mm)	21.59 ± 2.54	23.00 ± 2.61	1.41	<0.001 *	Paired *t*-test
N-S (mm)	59.50 ± 4.10	60.73 ± 5.21	1.23	0.018 *	Paired *t*-test
N-S/Ba-N (%)	73.37 ± 2.82	72.49 ± 2.91	−0.88	0.001 *	Paired *t*-test
Ba-S/Ba-N (%)	26.64 ± 2.82	27.52 ± 2.91	0.88	0.001 *	Paired *t*-test
Midface Depth and Ratios					
N-Me (mm)	100.53 ± 5.20	104.50 ± 6.14	3.97	<0.001 *	Paired *t*-test
N-Me/Ba-N (%)	124.08 ± 4.52	124.99 ± 5.52	0.92	0.2	Paired *t*-test
Ba-A/Ba-N (%)	98.48 ± 3.52	99.01 ± 4.58	0.53	0.357	Paired *t*-test
S-Ptm/Ba-N (%)	20.89 ± 3.82	20.39 ± 3.32	−0.49	0.287	Paired *t*-test
Ptm-A/Ba-N (%)	50.39 ± 3.04	52.13 ± 6.03	1.74	0.050 *	Wilcoxon test
Mandibular Morphology					
Gonial Angle (°)	128.94 ± 2.70	127.47 ± 3.27	−1.47	0.030 *	Wilcoxon test
Ba-Pog/Ba-N (%)	94.04 ± 8.08	91.96 ± 8.59	−2.08	0.003 *	Paired *t*-test
Ar-Pog/Ba-N (%)	82.99 ± 8.00	80.73 ± 8.32	−2.26	0.007 *	Paired *t*-test
Ba-Ar/Ba-N (%)	11.06 ± 2.11	11.25 ± 2.43	0.19	0.621	Paired *t*-test
Ar-Go/Ba-N (%)	9.66 ± 3.25	7.42 ± 4.01	−2.24	0.005 *	Paired *t*-test
Go-Pog/Ba-N (%)	73.35 ± 5.74	73.28 ± 5.69	−0.06	0.934	Paired *t*-test
Facial Height Ratios					
N-S/N-Me (%)	8.49 ± 1.95	7.44 ± 1.69	−1.06	0.049 *	Paired *t*-test
S-Ar/N-Me (%)	25.83 ± 2.04	25.38 ± 2.20	−0.45	0.342	Paired *t*-test
Ar-Go/N-Me (%)	36.85 ± 3.07	36.46 ± 3.20	−0.39	0.712	Paired *t*-test
S-Go/N-Me (%)	62.66 ± 2.77	61.85 ± 3.71	−0.81	0.163	Wilcoxon test
Anterior Facial Height					
N-ANS/N-Me (%)	45.04 ± 2.02	45.34 ± 1.97	0.3	0.451	Paired *t*-test
ANS-Me/N-Me (%)	54.61 ± 1.69	54.55 ± 1.83	−0.06	0.856	Paired *t*-test
ANS-U1/N-Me (%)	23.05 ± 2.09	22.63 ± 1.88	−0.42	0.42	Paired *t*-test
L1-Me/N-Me (%)	32.42 ± 1.33	32.40 ± 1.74	−0.02	0.956	Paired *t*-test
U1-L1/N-Me (%)	0.93 ± 2.23	0.74 ± 1.09	−0.19	0.463	Wilcoxon test

Note: SD, Standard Deviation; Diff., Mean Difference (T2−T1). Measurement units are mm unless specified as degrees (°) or percentage (%). Statistical significance is indicated by bold text and asterisk (* *p* < 0.05). N-Me (Nasion-Menton) represents anterior facial height.

**Table 2:** Comparison of Coben analysis measurements before (T1) and after (T2) RAMPA treatment in female patients (*n* = 13).

Category and Measurement	T1 Mean ± SD	T2 Mean ± SD	Mean Diff.	*p*-Value	Statistical Test
Cranial Base and Depth					
Ba-N (mm)	81.17 ± 10.31	83.35 ± 10.42	2.18	0.007 *	Paired *t*-test
Ba-S (mm)	22.42 ± 3.41	23.45 ± 3.31	1.03	0.074	Wilcoxon test
N-S (mm)	58.75 ± 7.76	59.89 ± 7.93	1.15	0.018 *	Paired *t*-test
N-S/Ba-N (%)	72.37 ± 2.33	71.81 ± 2.28	−0.55	0.204	Paired *t*-test
Ba-S/Ba-N (%)	27.60 ± 2.34	28.20 ± 2.27	0.6	0.195	Paired *t*-test
Midface Depth and Ratios					
N-Me (mm)	101.90 ± 13.27	105.54 ± 14.62	3.64	0.004 *	Paired *t*-test
N-Me/Ba-N (%)	125.60 ± 4.26	126.50 ± 5.00	0.9	0.42	Paired *t*-test
Ba-A/Ba-N (%)	97.30 ± 2.66	99.90 ± 3.63	2.6	0.001 *	Paired *t*-test
S-Ptm/Ba-N (%)	20.50 ± 1.80	19.60 ± 3.35	−0.9	0.267	Paired *t*-test
Ptm-A/Ba-N (%)	49.10 ± 2.79	52.30 ± 2.64	3.2	<0.001 *	Paired *t*-test
Mandibular Morphology					
Gonial Angle (°)	124.23 ± 5.85	123.00 ± 4.49	−1.24	0.261	Paired *t*-test
Ba-Pog/Ba-N (%)	95.80 ± 5.27	94.80 ± 5.27	−1	0.37	Paired *t*-test
Ar-Pog/Ba-N (%)	84.00 ± 6.57	83.50 ± 6.18	−0.5	0.628	Paired *t*-test
Ba-Ar/Ba-N (%)	11.80 ± 2.59	11.20 ± 2.46	−0.6	0.157	Paired *t*-test
Ar-Go/Ba-N (%)	8.00 ± 3.23	6.20 ± 3.14	−1.8	0.047 *	Paired *t*-test
Go-Pog/Ba-N (%)	75.90 ± 5.61	77.30 ± 5.05	1.3	0.056	Paired *t*-test
Facial Height Ratios					
N-S/N-Me (%)	7.70 ± 2.28	6.70 ± 3.22	−1	0.23	Wilcoxon test
S-Ar/N-Me (%)	25.40 ± 2.97	26.30 ± 3.12	0.9	0.037 *	Paired *t*-test
Ar-Go/N-Me (%)	40.70 ± 8.43	39.00 ± 9.85	−1.7	0.040 *	Paired *t*-test
S-Go/N-Me (%)	64.40 ± 4.68	63.20 ± 4.97	−1.2	0.108	Paired *t*-test
Anterior Facial Height					
N-ANS/N-Me (%)	45.40 ± 2.56	45.00 ± 2.07	−0.4	0.378	Paired *t*-test
ANS-Me/N-Me (%)	54.10 ± 1.75	54.80 ± 2.11	0.7	0.027 *	Paired *t*-test
ANS-U1/N-Me (%)	22.90 ± 2.14	23.00 ± 1.49	0.1	0.717	Paired *t*-test
L1-Me/N-Me (%)	32.50 ± 1.63	33.10 ± 1.78	0.6	0.156	Paired *t*-test
U1-L1/N-Me (%)	2.30 ± 2.70	1.40 ± 1.42	−0.9	0.187	Paired *t*-test

Note: SD, Standard Deviation; Diff., Mean Difference (T2−T1). Measurement units are mm unless specified as degrees (°) or percentage (%). Statistical significance is indicated by bold text and asterisk (* *p* < 0.05). N-Me (Nasion-Menton) represents anterior facial height.

**Table 3:** Descriptive comparison of craniofacial changes between the RAMPA treatment group and historical natural growth patterns.

Measurement Parameter	RAMPA Treatment Outcomes	Natural Growth Patterns (Mitani’s Study [29])	Comparative Observation and Implication
Facial Depth (Ba-N) and Height (N-Me)	Significant increases in both males and females (Anterosuperior growth).	Significant increases observed, but at a distinct rate/pattern compared to treatment.	Similarity: Both show anterosuperior growth Implication: RAMPA appears to accelerate or concentrate this growth tendency within the treatment period.
Maxillary Depth Ratio (Ptm-A/Ba-N)	Significant increase in males; Highly significant increase in females.	Absolute depth increases, but the ratio relative to cranial base tends to decrease or remain constant.	Difference: RAMPA induces active maxillary protraction, increasing the maxilla’s relative contribution to total facial depth, unlike natural growth.
Gonial Angle	Significant decrease in males (Anterior rotation).	Growth trajectory varies; often linear or slight closure, but less pronounced.	Difference: RAMPA induces a clearer counter-clockwise rotation (horizontal growth vector) compared to the natural tendency.
Mandibular Ratios (Ar-Go/Ba-N, etc.)	Significant proportional decrease (reflecting rotational rearrangement).	Ratios may change due to size increase, but rotational component is less dominant.	Difference: RAMPA emphasizes 3D rearrangement via anterior rotation, altering Coben’s proportional ratios more distinctly.

**Table 4:** Major Craniofacial Structural Changes and Clinical Implications with RAMPA Treatment.

Measurement	Gender	Direction of Change	Average Change/Pattern	*p*-Value	Clinical/Biomechanical Implication
Ba-N	Male	Increase	T2 > T1 (average 2.65 increase)	0.00095	Induction of anterosuperior growth, increased facial depth
N-Me	Male	Increase	T2 > T1 (average 3.97 increase)	0.00007	Induction of anterosuperior growth, increased anterior facial height
Ptm-A/Ba-N	Male	Increase	T2 median > T1 median	0.04983	Anterior growth of maxilla and proportional increase in depth
Gonial Angle	Male	Decrease	T1 median > T2 median	0.03024	Induction of anterior mandibular rotation and horizontal growth
Ar-Go/Ba-N	Male	Decrease	T1 > T2 (average 2.24 decrease)	0.00465	Proportional rearrangement due to anterior mandibular rotation
Ar-Pog/Ba-N	Male	Decrease	T1 > T2 (average 2.26 decrease)	0.00743	Proportional rearrangement due to anterior mandibular rotation
Ba-Pog/Ba-N	Male	Decrease	T1 > T2 (average 2.08 decrease)	0.00327	Proportional rearrangement of lower facial depth due to anterior mandibular rotation
N-S/N-M	Male	Decrease	T1 > T2 (average 1.06 decrease)	0.04929	Proportional change between cranial base and facial height, potential for posture improvement
Ba-N	Female	Increase	T2 > T1 (average 2.18 increase)	0.00711	Induction of anterosuperior growth, increased facial depth
N-S	Female	Increase	T2 > T1 (average 1.15 increase)	0.01833	Induction of anterosuperior growth, increased anterior cranial base length
N-Me	Female	Increase	T2 > T1 (average 3.64 increase)	0.00430	Induction of anterosuperior growth, increased anterior facial height
Ptm-A/Ba-N	Female	Increase	T2 > T1 (average 3.20 increase)	0.00053	Strong anterior growth of maxilla and proportional increase in depth
Ba-A/Ba-N	Female	Increase	T2 > T1 (average 2.64 increase)	0.00131	Strong growth of midface and proportional increase in depth
Ar-Go/Ba-N	Female	Decrease	T1 > T2 (average 1.76 decrease)	0.04684	Proportional rearrangement due to anterior mandibular rotation
S-Ar/N-M	Female	Increase	T2 > T1 (average 0.86 increase)	0.03681	Contribution to height growth of posterior–superior face
Ar-Go/N-M	Female	Decrease	T1 > T2 (average 1.73 decrease)	0.04005	Proportional rearrangement due to anterior mandibular rotation

## Data Availability

The data underlying this article will be shared on reasonable request to the corresponding author.

## Abbreviations

FH	Frankfort Horizontal plane
RAMPA	Right Angle Maxillary Protraction Appliance
Ba-N	Basion to Nasion (Cranial base length)
Ba-A	Basion to Point A (Total maxillary depth)
Ba-S	Basion to Sella (Posterior cranial base length)
S-Ptm	Sella to Pterygomaxillary fissure
Ptm-A	Pterygomaxillary fissure to Point A (Maxillary unit length)
Ba-Ar	Basion to Articulare
Ar-Go	Articulare to Gonion (Ramus height/width)
Go-Po	Gonion to Pogonion (Mandibular body length)
R∠	Ramus inclination
G∠	Gonial angle
NM (N-Me)	Nasion to Menton (Total anterior facial height)
N-S	Nasion to Sella vertical distance
S-Ba	Sella to Basion vertical distance
S-Ar	Sella to Articulare vertical distance
N-ANS	Nasion to Anterior Nasal Spine (Upper anterior facial height)
ANS-Me	Anterior Nasal Spine to Menton (Lower anterior facial height)
ANS-1	ANS to Upper Incisor edge
1-1	Overbite
M-1	Menton to Lower Incisor edge
